# Research Progress of CLE and Its Prospects in Woody Plants

**DOI:** 10.3390/plants14101424

**Published:** 2025-05-09

**Authors:** Zewen Song, Wenjun Zhou, Hanyu Jiang, Yifan Duan

**Affiliations:** 1 Co-Innovation Center for Sustainable Forestry in Southern China, College of Life Sciences, Nanjing Forestry University, Nanjing 210037, China; szw1103936553@163.com (Z.S.); 15850592987@163.com (W.Z.); 2 College of Forestry and Grassland, Nanjing Forestry University, Nanjing 210037, China; 17786523492@163.com

**Keywords:** CLE family, meristem, regulatory networks, compensation mechanism, plant development

## Abstract

The peptide ligands of the CLAVATA3/EMBRYO SURROUNDING REGION-RELATED (CLE) family have been previously identified as essential signals for both short- and long-distance communication in plants, particularly during stem cell homeostasis, cell fate determination, and growth and development. To date, most studies on the CLE family have focused on model plants and especially those involving stem and apical meristems. Relatively little is known about the role of CLE peptides in tall trees and other plant meristems. In this review, we summarize the role of *CLE* genes in regulating plant Root Apical Meristem (RAM), Shoot Apical Meristem (SAM), Procambium, Leaf and Floral Meristem (FM), as well as their involvement in multiple signaling pathways. We also highlight the evolutionary conservation of the *CLE* gene family and provide a comprehensive summary of its distribution across various plant developmental tissues. This paper aims to provide insights into novel regulatory networks of CLE in plant meristems, offering guidance for understanding intercellular signaling pathways in forest trees and the development of new plant organs.

## 1. Introduction

The growth of trees originates from the development of embryonic stem cells within the seed. When plants have fully matured and sensed the appropriate external conditions, the differentiation and division of embryonic stem cells are precisely and stably regulated, which is the basis for the formation and maintenance of meristem tissues for sustaining development of plants after embryo [1,2]. The radicle firstly divides into RAM, and with the elongation of the plumular axis, the germ differentiated SAM to form stems and leaves, and then the procambium cells continued to increase differentiation. Eventually, the plant transitions from vegetative growth to reproductive development, marked by the formation of the FM [3,4,5]. In recent years, many genes have been concerned with the regulation of plant meristem, such as *FASCIATA1* and *FASCIATA2* (*FAS1*/*FAS2*), *SHOOT MERISTEMLESS* (*STM*), and *CLAVATA3* (*CLV3*). Among these, the *CLE* gene family has been extensively studied as key genes [6,7,8,9,10].

The *CLE* family of plant-specific genes is named after its founding *CLV3*/*ESR* gene that is specifically expressed in *maize* (*Zea mays*) [11,12,13]. Furthermore, Cock and McCormick discovered 39 related protein sequences associated with the CLV3/ESR family, which they named the CLE family. These proteins were characterized by conserved 12-residue domains essential for ensuring the function of C-terminal signal peptides and N-terminal hydrophobic signaling peptide [11]. The similarity of the remaining sequences is very low except for the conserved motif and secreted signal peptide [14]. Subsequently, CLE is explicitly described as a signal peptide that is cleaved from a longer pre-peptide with a similar structure: small proteins (usually fewer than 150 amino acids) consisting of an N-terminal signal peptide, followed by a variable domain with significant sequence diversity, and a conserved C-terminal CLE motif. These pre-peptides are translated and modified one or more times [11,14,15,16]. As for *CLE* gene family function studies, as early as the mid-1990s, most *CLV1*/*CLV3* mutants were found to affect the meristem activity of plant stems, roots, and flowers [17,18]. Subsequently, the WUSCHEL (WUS)-CLV3 regulatory network was discovered, which controls the activity of the apical meristem at the stem tip [19]. In 2002, CLV3-CLV1/CLV2 was found as a receptor ligand in plants to signal from the stem cell population [20]. This marked the beginning of further studies on the CLE family. However, this is only applicable to the *CLV1*/*CLV2*/*CLV3* genes. In 2006, CLV3/ESR1-LIKE 41 (CLE41) was shown to repress xylem differentiation in cell culture [21]. In a later study, similar to WUS and CLV3, CLE40 and WUS-RELATED HOMEOBOX 5(WOX5) were found to play a role in regulating the root meristem [22,23,24]. As the research on various genes of the *CLE* family has been continuously deepened, it has been discovered that *CLE* gene family have different functions to control the development of plants.

Based on domain structure and functional analyses, Whitford classified the peptide types of the CLE family into two categories: A (CLAVATA3 (CLV3)-like) and B (TRACHEARY ELEMENT DIFFERENTIATION INHIBITORY FACTOR (TDIF)-like) [25]. A-type CLE peptides promote cell differentiation in root and shoot apical meristems, whereas the B-type peptides CLE41–CLE44 do not promote. B-type CLE peptides suppress differentiation into tracheary elements. B-Type CLE peptides are mainly Tracheary Element Differentiation Inhibitory Factor TDIF-like [26,27]. The synergistic interaction of these two peptides inhibits differentiation and promotes auxin-mediated cell proliferation in the secondary meristem (vascular cambium), suggesting that specific *CLE* genes have dual functions and cell type-specific responses [28].

At present, the research on woody plants mainly focuses on the genetic transformation system [29], the molecular regulation related to wood formation [30,31,32], the molecular basis of forest economic traits [33,34], and the response control of plant stress resistance [35,36]. While the *CLE* gene family as a systemic regulatory hub governing stem cell dynamics across *Arabidopsis* thaliana tissues—particularly during organogenesis from embryogenesis to post-meristematic differentiation—the functional characterization of CLE networks in woody perennials remains critically understudied. The earliest research on *CLE* genes in forest trees was reported in *Populus trichocarpa* in 2016 [37]. Since then, research on the *CLE* gene family has focused on identifying the systematic classification of its family in woody plants or verifying a single function: for example, *Camellia oleifera* and some rosaceous plants, involving very few molecular regulatory networks [38,39]. Only *Populus trichocarpa* has been gradually studying the CLE family regulatory network during the development of vascular tissues, but the progress has been slow. Forest trees play a crucial role in water resource conservation, maintaining ecological balance, and providing medicinal compounds from their roots and leaves along with edible fruits, making them indispensable for both environmental sustainability and human well-being. For tall trees to grow healthily and vigorously, it is essential to maintain and properly differentiate the stem cells in the meristematic tissues. Here, we investigate the regulatory pathway of the CLE family by tracing the developmental sequence of plant organ formation, aiming to offer guidance for the growth and development of forest trees.

## 2. Development and Maintenance of CLEs in Root Apical Meristem

**Figure 1:**
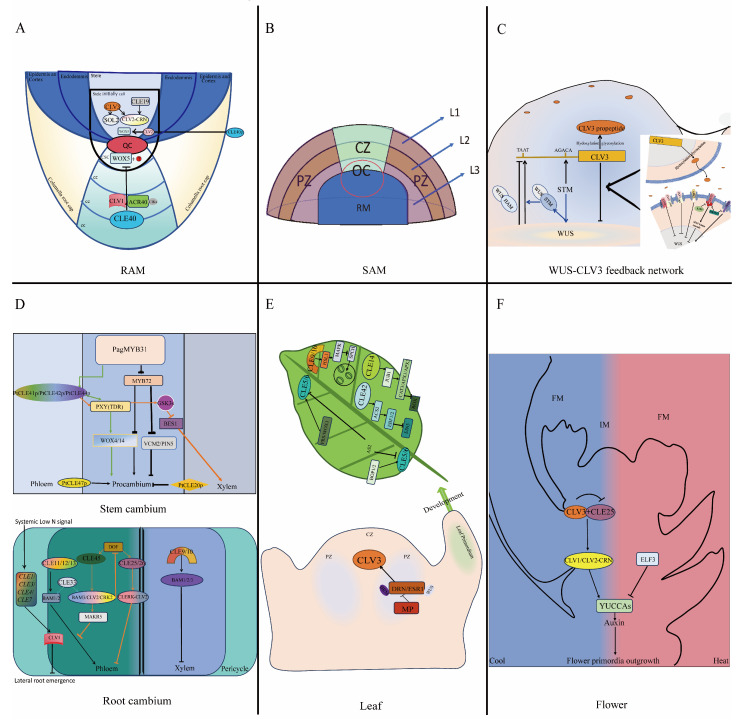
The regulatory network map of the CLE family in plant meristems. (**A**) Summary of the mechanism model of root cell maintenance and differentiation in the root mediated by the CLE family. Thick line segment: SCN; Arrow: Ligand reception/receptor activation; Blunt arrow: inhibition (Refer to [24]). (**B**) The *Arabidopsis* SAM is divided into L1/epidermis, L2/sub-epidermis and Corpus/L3. The same *Arabidopsis* SAM is divided into distinct zones, including CZ, PZ, OC, and RM (Refer to [46]). (**C**) WUS-CLV3-STM regulatory circuits involve peptide hormones and receptor kinases in SAM. In the rectangular box of the picture, the CLV3-WUS regulatory pathway is drawn. Arrow: Ligand reception/receptor activation; Blunt arrow: inhibition (Refer to [47]). (**D**) CLE family transcription control and receptor–ligand signaling are involved in the balance between procambium, phloem, and xylem maintenance in stem and root. Different colored lines are used to distinguish the different regulatory pathways involving the same receptor. Arrow: Ligand reception/receptor activation; Blunt arrow: inhibition. (**E**) The regulatory pathways of CLE family during the initiation and development of leaf primordia. Green thick arrow: The prothallus develops into a leaf. Arrow: Ligand reception/receptor activation; Blunt arrow: inhibition. (**F**) The CLV3-CLV1/CLV2-CRN pathway interacts with ambient temperature, acting on auxin synthesis and controlling the growth of flower primordium at different temperatures. Blue indicates low temperature while red represents high temperature. Arrow: Ligand reception/receptor activation; Blunt arrow: inhibition (Refer to [48]).

Root developmental plasticity is a critical determinant of plant fitness, enabling efficient acquisition of soil resources (water and nutrients) and systemic coordination of whole-plant growth. The establishment of RAM architecture begins with a stereotypical radial pattern at the root tip, where a small cohort of progenitor cells undergoes precisely oriented divisions to generate distinct tissue lineages [40]. Central to this process is the stem cell niche (SCN), a dynamic microdomain organized around the mitotically inactive quiescent center (QC) (Figure 1A). This niche contains the QC that is thought to be the initial cell that maintains the first surrounding cell in an undifferentiated state and gives rise to other stem cells; stem cells on the proximal (toward the shoot) side of the QC generate vasculature and pericycle; lateral stem cells of the QC give rise to endodermis, cortex, epidermis, lateral root cap; and distal columella stem cells (CSC) of the QC generate the protective cap of columella cells (CC) [41,42,43,44,45].

Unlike stems, root ecological niche restriction is mediated not by individual stem cells but by entire meristematic stem cell populations enveloped by the root cap [24,49]. CLE40 expression is localized to the basal region of the embryo during the globular embryo stage, where it initiates root meristem and vasculature formation. Post-germination, CLE40 is expressed in the CCs and localized at the distal end of QC [24,50]. Through the receptor-like kinase CLV1 and the CIKs (CLAVATA3 INSENSITIVE RECEPTOR KINASES)-assisted ARABIDOPSIS CRINKLY4 (ACR4) [51], CLE40 limits QC-derived signals whose activity or expression might depend on WOX5 function in the QC and act non-cell-autonomously to control CSC fate. CLE40 can positively regulate the promotion of WOX5 expression via the CLV2 receptor in the vascular initials [52]. In *Solanum tuberosum*, the homologous gene of *CLV3* is *StCLE4*, which regulates stem cell activity and modulates both stem and root growth [53]. In lateral root apex meristem activity, *CLV3* plays a central role in lateral root apical meristem activity. Under normal conditions, *CLV3* is expressed in the pericycle of roots, and lateral root length is inversely correlated with *CLV3* expression levels. However, *CLV3* overexpression disrupts root tip meristem activity, leading to a short-root phenotype that is positively influenced by sucrose levels in the root [54]. Additionally, the CLV3-CLV2/SOL pathway regulates root meristem signaling, with SUPPRESSOR OF LLP1 2 (SOL2)/CORYNE (CRN) deficiency resulting in markedly reduced root length [55,56] (Figure 1A). Overexpression of *CLE19* restricts root meristem cell size without directly affecting the QC or adjacent stem cells, instead acting on pericycle initiation cells via the CLV2 complex [57,58].

## 3. Development and Maintenance of CLEs in Shoot Apical Meristem

In forest trees, almost all above-ground tissues originate from the conserved dome-like SAM, which is actually a highly heterogeneous and highly organized structure controlled by stem cells [59,60]. Structurally, in monocotyledons like *Oryza sativa* L., SAM is organized into distinct layers: the L1 layer and L2 layer [61,62]. In dicotyledons, SAM forms between the two cotyledons and comprises three stem cell layers: the L1 layer, which generates the epidermis via anticlinal divisions; the L2 layer where cells undergo periclinal divisions in the meristem and produce mesophyll cells through vertical/peripheral divisions in leaf primordia; and the L3 layer, which differentiates into stem cell centers and vascular tissues via both anticlinal and periclinal divisions, as seen in *Arabidopsis thaliana* [59,63]. Functionally, stem cells organize the SAM into three domains: Central Zone, (CZ): including the organizing center (OC) which a central domain with slow division rates that maintains meristem integrity and supplies cells to the peripheral zone (PZ); the PZ, surrounding the OC, where rapid cell divisions generate organ primordia; the rib meristem (RM), located below the OC, which specifies central stem cell identity [64,65] (Figure 1B). Cells continuously proliferate, progressing through three cellular functional regions that are aimed to control different cells and, thus, regulate the differentiation, division, and formation of organ primordia and internal tissues [65].

*CLV3* is localized to the overlying cell layers of the stem cell niche, where it regulates cell division and organogenesis, while *WUS*, which specifies stem cell identity and controls meristem activity, resides at the base of the stem tip [23,63,66]. *STM*, which maintains stem cell pluripotency, works synergistically with WUS and CLV3 to form a WUS-CLV3-STM regulatory loop that governs SAM homeostasis [67,68]. During the proliferation and differentiation of SAM stem cells, a Homeobox (HB) family transcription factor WUS and a Class I KNOX transcription factor STM can upregulate *CLV3* expression by binding to the *CLV3* promoter cis-acting elements (TAAT and TGACA), respectively [69,70]. Furthermore, STM assists WUS in forming WUS-STM heterodimers, which enhance WUS binding affinity to the *CLV3* promoter via protein–protein interactions. This promotes *CLV3* expression in the central zone (CZ), ensuring stem cell population stability [69,70,71]. The *CLV3* gene encodes a 96-amino acid precursor protein that is post-translationally modified to yield a mature arabinosylated glycopeptide. This peptide contains a conserved 12- to 13-amino acid motif, with Leu and Arg identified as critical residues for restricting SAM size [11,66,72,73,74]. Spatiotemporally, *CLV3* expression is confined to the epidermal and subepidermal layers of the CZ in shoot and floral meristems but is absent in the RM [66,67,70] (Figure 1C).

When *CLV3* promotes cell proliferation, elevated WUS levels recruit HAIRY MERISRTEM1/2 (HAM1 and HAM2 (members of the GRAS transcription factor-encoding HAM family)). These WUS-HAM heterodimers suppress *CLV3* expression, thereby establishing apical polarity of the *CLV3* expression domain along the SAM axis to regulate stem cell homeostasis [46,75,76,77,78,79]. In embryonic development, *CLV3* expression is regulated exclusively by WUS, independent of STM [80]. However, during later developmental stages, STM and WUS jointly modulate CLV3 levels, with CLV3 responsiveness to WUS confined to the apical meristem. Sustained overexpression of *WUS* triggers exocytosis-dependent CLV3 signaling, which coordinates four distinct cellular pathways to repress WUS in the RM, forming a negative feedback loop [20,81]. Mechanistically, CLV3 inhibits *WUS* primarily via LEUCINE-RICH REPEAT RECEPTOR-LIKE KINASES (LRR-RLKs) and CIKs. These receptors act synergistically, where CIKs enhance LRR-RLK activity to amplify downstream signaling cascades that suppress *WUS* expression [74,82,83] (Figure 1C).

### 3.1. CLV3-CLV1

As a ligand–receptor pair, CLV3 undergoes proteolytic cleavage and directly binds to CLV1, an LRR-RLK, with a dissociation constant (Kd) of 17.5 nM. This interaction triggers CLV1 endocytosis to regulate its membrane trafficking [84,85,86]. The binding ability between CLV3 and CLV1 is mainly affected by the arabinosylation of CLV3 and the affinity of different amino acids in the extracellular domain of CLV1 [84,86,87,88] (Figure 1C).

### 3.2. CLV3-CLV2-CRN/SOL2

Both CLV2, a LEUCINE-RICH REPEAT (LRR, protein lacking a kinase domain) and SUPPRESSOR OF LLP1 2 (SOL2)/CORYNE (CRN) (a transmembrane pseudokinase devoid of LRRs) are synthesized on the endoplasmic reticulum (ER). The transmembrane (TM) domain of CRN binds specifically to CLV2, enabling the CRN-CLV2 complex to localize to the plasma membrane (PM). This interaction neutralizes an acidic inhibitory motif in the extracellular region of CLV2, which is essential for PM trafficking. Notably, CRN does not enhance CLV2 accumulation at the PM but facilitates its targeting. The mature CRN-CLV2 complex subsequently binds CLV3 to mediate signaling [84,89,90,91]. CLV2-CRN is parallel to CLV1 and co-responds to CLV3 signal transduction [92,93] (Figure 1C).

### 3.3. CLV3-RPK2

As a member of the RLKS family of receptor-like kinases, (RECEPTOR-LIKE PROTEIN KINASE2 (RPK2))/TOAD2 regulates the development of anther microspores and tapetum, and mutations in *RPK2* cause anther breakage [94,95]. In *CLV3*-null backgrounds, *RPK2* mutants exhibit reduced SAM size and increased carpel number, indicating that RPK2 participates in CLV3-dependent signaling within the SAM to repress *WUS* expression. While RPK2 does not directly bind CLV3 via its leucine-rich repeat (LRR) domain, the mechanism of interaction remains unclear [96]. We hypothesize that RPK2 participates in the CLV3 pathway not solely as a ligand but may act through alternative mechanisms in plant signaling (Figure 1C).

### 3.4. CLV3-BAMs

BARELY ANY MERISTEM(BAM) is one of the leucine-rich repeat receptor-like kinases (LRR-RLK). Constitutive expression of *BAM1*/*BAM2* partially rescues the *CLV1* mutant phenotype, confirming their functional homology with *CLV1*. Unlike *CLV1*, *BAM1*/*BAM2* exhibit broader expression patterns [97]. Photoaffinity labeling assays demonstrate direct binding between the BAM1 ectodomain and CLV3 peptide. While single mutants (*BAM1*, *BAM2*, or *BAM3*) show no obvious developmental defects, double (*BAM1*/*BAM2*) and triple *(BAM1*/*BAM2*/*BAM3*) mutants exhibit reduced SAM size due to stem cell depletion [96,98] (Figure 1C). Thus, the CLV3-BAMs regulatory pathway was identified.

Current understanding of CLV3 downstream signaling—particularly phosphorylation cascades involving kinases and phosphatases—remains limited. CLV3 activates a phosphorylation cascade mediated by MPK3/MPK6, which partially rescues the *CLV1* mutant phenotype, implicating these kinases in dependent signaling CLV1 [99]. The kinase-associated protein phosphatase KAPP directly interacts with CLV1, dephosphorylating it to attenuate CLV1 activity. Additionally, the PP2C-type phosphatases POLTERGEIST (POL) and POL-LIKE1 (PLL1) act as negative regulators downstream of CLV1/BAM receptors, modulating *WUS* expression to control apical stem cell dynamics [83,100] (Figure 1C). Genetic evidence shows that CLV3-CLV1, CLV3-CLV2/SOR function independent of each other. However, studies on pathway crosstalk show that the first two pathways may be connected these pathways may converge-potentially compensating for each other to form a regulatory network that maintains stem cell homeostasis when one pathway is dysregulated [82].

## 4. Development and Maintenance of CLEs in Stem and Root Cambium

Stem and root apical meristem cells continue to divide and differentiate to form the procambium that have tissue with permanent meristematic activity. Procambium serves as the primary source of xylem and phloem cells, while also contributing to the structural framework of plant stems and roots [101].

In the stem, shoot apical localized procambium (PC) initials are described as the primary meristem that differentiates basally to produce primary vascular bundles that daughter cells of PC differentiate into protophloem (PPh) toward the outside of the stem and protoxylem (PXy) toward the inside of the stem. Moving basally toward developmentally older tissues, actively dividing meristematic cells within vascular bundles were described as metacambium (MC) that subsequently divide into the secondary vascular cambium meristematic cells that produce secondary phloem and secondary xylem [102,103,104]. In this review, we classify stem tissues into procambium, xylem, and phloem to elucidate CLE family-mediated regulatory mechanisms. Within the procambium, the TDIF predominantly regulates cambial activity [25]. In *Populus trichocarpa*, MYB31 located in the cambium layer regulates the PtCLE41/PtCLE42/PtCLE44 peptides produced by the phloem to translocate the cambium and through the TDIF RECEPTOR (TDR)/PHLOEM INTERCALATED WITH XYLEM (PXY) membrane protein kinase signaling pathway, the PtCLE41p/PtCLE42p/PtCLE44p combine with *WOX4*/*WOX14* to promote procambial cell proliferation while suppressing xylem cell differentiation [105,106]. As a downstream transcription factor of TDIF-PXY, GLYCOGEN SYNTHASE KINASE 3 PROTEINS (GSK3s) inhibit BRI1-EMS SUPPRESSOR 1 (BES1), thereby inhibiting cambium-to-xylem cell differentiation [107]. In *Populus trichocarpa*, MYB31 could either promote cell proliferation through restraining the MYB31-MYB72-WOX4 module or inhibit cambial activity through restraining the MYB31-MYB72-VASCULAR CAMBIUM-RELATED MADS 2 (VCM2)/PIN-FORMED 5 (PIN5) modules (VCM2/PIN5) [108,109]. In gymnosperms, *CLE41*/*CLE44* play a role not only in the phloem but also in the tracheary elements (TEs) [105,106]. PtCLE47 and PtCLE20, two poplar CLE polypeptides, respectively, promote and inhibit procambial cell proliferation [110,111] (Figure 1D).

In the root, the procambium can generate primary xylem and primary phloem which includes sieve elements (SEs), companion cells (CCs) and related cell types. The pericycle generates lateral roots and initiates vascular cambium (responsible for secondary phloem and xylem production) [47,112]. In xylem precursor cells, the receptor-like kinases BAM1, BAM2, and BAM3 collectively function as major receptors for CLE9/CLE10 peptides, negatively regulating periclinal cell division to control xylem file numbers [21,113] (Figure 1D). During protophloem development, *CLE33* critically modulates BAM1/BAM2/BAM3 and CLV2/CRN complexes to regulate SE differentiation [114]. In the protophloem, *CLE25*/*CLE26* are expressed early in the SE cells lineage and promote the initiation and development of phloem through the complex interaction with CLE-RESISTANT RECEPTOR KINASE-CLV2 (CLERK-CLV2) receptor to control the SE precursor cell (SPC) receptor-like protein [41,115,116]. A suppressor screen of *BREVIS RADIX* (*BRX*) mutants identified the CLE45-BAM3 axis as a compensatory pathway for SE differentiation [117]. MEMBRANE-ASSOCIATED KINASE REGULATOR 5 (MAKR5) acts as a post-transcriptionally regulated amplifier of the CLE45p signal that acts downstream of BAM3 [118,119]. However, this way of signaling antagonizes BAM1/BAM2-mediated CLE11/CLE12/CLE13 signaling in the phloem initials [119]. Additionally, phloem-Dofs not only enforce SE and CC formation but also activate the production of CLE25, CLE26, and CLE45 that reduce the level of phloem-Dofs by interacting with BAMs/CIKs, thereby inhibiting the excessive production of SEs and CCs [120]. Furthermore, CLE peptides (CLE1/CLE3/CLE4/CLE7) modulate lateral root growth and branching through the CLE-CLV1 signaling module in response to nitrogen availability, without affecting primary root development [121]. Collectively, these pathways fine-tune root architecture and elongation (Figure 1D).

## 5. Development and Maintenance of CLEs in Leaf

Leaf initiation and proper spatial orientation are essential for efficient photosynthesis, thereby ensuring plant survival. Within the SAM, the CZ harbors stem cells, while organogenesis initiates in the PZ [122]. During vegetative SAM development, CZ-derived stem cells undergo continuous division, with daughter cells migrating laterally into the PZ to form leaf primordia structures that are small and regularly spaced [123]. Cells in the PZ region divide rapidly and continuously, forming leaf protodermal cells, which can either directly divide into pavement cells (general epidermal cells) or become meristemoid mother cells (MMCs) that are stomatal lineage stem cells [124,125]. Following primordium initiation, leaves develop along three distinct polarity axes: axial-dorsal, proximal-distal, and central-lateral [126,127] (Figure 1E).

Auxin determines the fate of organ primordia in the peripheral region of PZ, and the formation of leaf primordia is dependent on the auxin maximum formed by the polar auxin transport mediated by the *PIN-FORMED 1* (*PIN1*) gene [128,129]. Belonging to AUXIN RESPONSE FACTORs (ARFs), ARF5 (Mp) shows threshold expression in PZ to CZ and mediates auxin signaling by negatively regulating CLV3 by repressing ENHANCER OF SHOOT REGENERATION1/DORNROSCHEN (ESR1/DRN) that can combine BRAHMA(BRM) and WUS to form a ternary protein complex [80,130,131,132,133,134,135,136]. This mode can prevent the axillary meristem (AM) disturbance caused by high expression of *CLV3*. In *Oryza sativa*, *NDL1* is the ortholog of *Arabidopsis thaliana* of *ESR1*/*DRN* and autonomously regulates leaf development [137]. This suggests that *CLV3* affects the development of leaf initially, and *CLE5*/*CLE6* are positively regulated by BLADE-ON-PETIOLE1/2 (BOP1/BOP2) at the petiole base so that their loss of function makes the petiole slightly wider. The transcription of *CLE5*/*CLE6* is negatively regulated by ASYMMETRIC LEAVES2 (AS2) at the distal positions of petioles and leaves. But CLE5/CLE6 have little effect on the leaf. Referring to the CLE-WOX pathway in SAM, it was found that the expression of *CLE5*/*CLE6* in leaves is also positively regulated by the WOX transcription factors, PRESSED FLOWER (PRS) and WOX1, which promote leaf growth and increase leaf margin cell-files [138]. In MCCs, CLE9/CLE10 bind to HAESA-LIKE1 receptor kinase (HSL1) to phosphorylate SPCH through a MAPK cascade to negatively control epidermal division [113]. By suppressing THE ENZYME 1-AMINOCYCLOPROPANE-1-CARBOXYLIC ACID SYNTHASE (ACS), CLE42 accumulates ETHYLENE-INSENSITIVE3 (EIN3)-binding F-BOX1/2 (EBF1/EBF2) protein, which degrades EIN3 (a master transcription factor in the ethylene pathway), a key component of the ethylene signaling pathway, through the ubiquitin–proteasome pathway, thereby delaying leaf senescence [139]. Additionally, CLE14 regulates age-dependent and stress-induced leaf senescence through promoting the expression of the JUB1-ROS scavenging gene (*CAT3*, *APX1*, *APX3*) to mediate ROS scavenging [139,140,141] (Figure 1E).

## 6. Development and Maintenance of CLEs in Floral Meristem

When the plant’s internal organs mature under a favorable external environment, SAM receives the flowering signal and transforms into the inflorescence meristem (IM), which marks the transition from vegetative growth to reproductive growth and then the formation of young flower primordia [142]. The young floral primordia retain apical stem cells that undergo lateral divisions within the IM, generating FMs. Each FM orchestrates the sequential development of floral organ whorls (sepals, petals, stamens, and carpels) to form a complete flower [143,144]. Floral organogenesis proceeds through a temporally and spatially regulated sequence, with partially overlapping phases ensuring precise whorl patterning [145]. Therefore, FMs are continuously produced by multiple developing organs, and unlike the SAM, which maintains expansive growth zones, FM activity occurs within spatially confined regions separated by narrow developmental boundaries [146].

*CLV2* is expressed in IM, and *CRN* is expressed in the early flower primordium and even expresses in the whole flower primordia. The CLV2/CRN receptor complex promotes the growth and development of flower primordia [147]. Mutations in the *CLV2* site lead to enlargement of stem and flower meristem and developmental defects in pistil, petals, and stamens [148]. *CLV1* and *CLV3* are expressed in the center and apex of FM. Compared with STM, *CLV1*/*CLV3* has the same expression pattern but opposite function; that is, *STM* mutants fail to form undifferentiated cells in stem meristem during meristem development while *CLV1/CLV3* mutants accumulate excessive undifferentiated cells in flower meristems, causing over proliferation of central floral tissues [17,63]. *STM* and *KNAT-6* mutations have additive effects in regulating CLV3 inflorescence size [19,149]. In *maize*, the *THICK TASSEL DWARF* (*TD1*) and *FASCIATED EAR2* (*FEA2*) genes encode CLV1-like LRR receptor kinase and the CLV2-like LRR receptor protein, respectively [150]. The *TD1* and *FEA2* double mutant exhibited a phenotype with an increased inflorescence size [151]. In *Oryza sativa* L, *FON1* encodes a gene homologous to *CLV1* and maize *TD1*, while *FON2* encodes a CLE protein associated with *AtCLV2*. In *FON1* and *FON2* mutants, FMs are increased, resulting in an increased number of flower organs such as stamens and carpels [152]. This suggests that *CLV1*/*CLV2*/*CLV3* genes affect flower development. Notably, *WUS* is not involved in floral meristem development and the CLV3-CLV1 regulatory pathway.

If the external environment temperature changes, *CLE* gene family, combined with auxin, play an irreplaceable role in responding to changes in flower primordia development [83]. At normal temperatures, the CLV3 pathway, like the thermal sensing *ELF3* factor containing the Poly-Q structure, is functionally degraded by being sequestered by the *YUCCA* (*YUC*) complex [153,154,155]. Under lower temperatures, receptor complexes CLV1 and CLV2/CRN transduce the *CLV3*/*CLE25* signal to promote normal flowering in plants by upregulating YUC-dependent auxin biosynthesis [48,156,157]. Although *CLE25* is inhibited by *CLV3*, in the case of *CLV3* mutation, *CLV3* promoter can bind to *CLE25* to compensate for the flower phenotype [157,158]. This suggests that the *CLV3* promoter pathway is shown to be important in regulating the transition state of flower primordia during vegetative-to-reproductive growth, though the intermediate pathways and associated genes remain uncharacterized. Under high temperatures, *ELF3* upregulates auxin to control flower development [48,155]. Therefore, the significance of temperature in regulating the CLE channel has also been given due attention [159] (Figure 1F).

## 7. Compensation Mechanism of CLEs in Plant

The compensation mechanism provides fault tolerance for plant development, enabling the maximization of growth along normal developmental trajectories [160,161,162]. Due to lineage-specific factors, the number, functional relationships, homologous retention, and diversity variation (including redundancy) of inbred family members differ significantly among distantly related species. However, the CLE protein family demonstrates remarkable structural conservation—particularly in the C-terminal CLE motif, which is critical for receptor binding [163]. In *Arabidopsis thaliana*, following *CLV3* deletion, the *CLE16* and *CLE17* signaling pathways actively regulate WUS, limiting stem and floral stem cell accumulation and buffering infinite apical enlargement caused by *CLV3* loss. These pathways are not sensed by CLV1 or CLV2 but exclusively by the BAM1/BAM2 receptor kinases, indicating their role as compensatory mechanisms for *CLV3* deficiency [97,164]. In *CLV1* mutants, ectopic *BAM* expression in the RM partially compensates for CLV1 loss [96,165,166]. Additionally, other CLE peptides may exhibit functional redundancy during SAM maintenance. This is evidenced by complete or partial *CLV3* complementation when *CLE1*, *CLE6*, *CLE9*, *CLE11*, *CLE12*, *CLE13*, *CLE19*, *CLE21*, or *CLE22* are expressed under the *CLV3* promoter [47,167]. Notably, single and double mutants of *CLE16*, *CLE17*, and *CLE27* show no detectable phenotypes in the SAM or IM.

In *Solanum lycopersicum*, *SlCLE* compensation is functionally active, with *SlCLE9* partially restoring *SlCLV3* stem cell homeostasis primarily via *SlCLV1* [158]. However, in *Arabidopsis thaliana*, the CLE9-CLV1 regulatory pathway remains poorly characterized. CLE40, encoding a putatively secreted protein with functional similarity to CLV3, can fully substitute for CLV3 in the SAM. The *CLV3* promoter drives *CLE40* expression to compensate for CLV3 deficiency [47]. In *Zea mays* L., *ZmFCP1* and *ZmCLE1E5* partially rescue the enlarged inflorescence meristem phenotype caused by *ZmCLE7* mutations [168].

## 8. Conclusions

**Figure 2:**
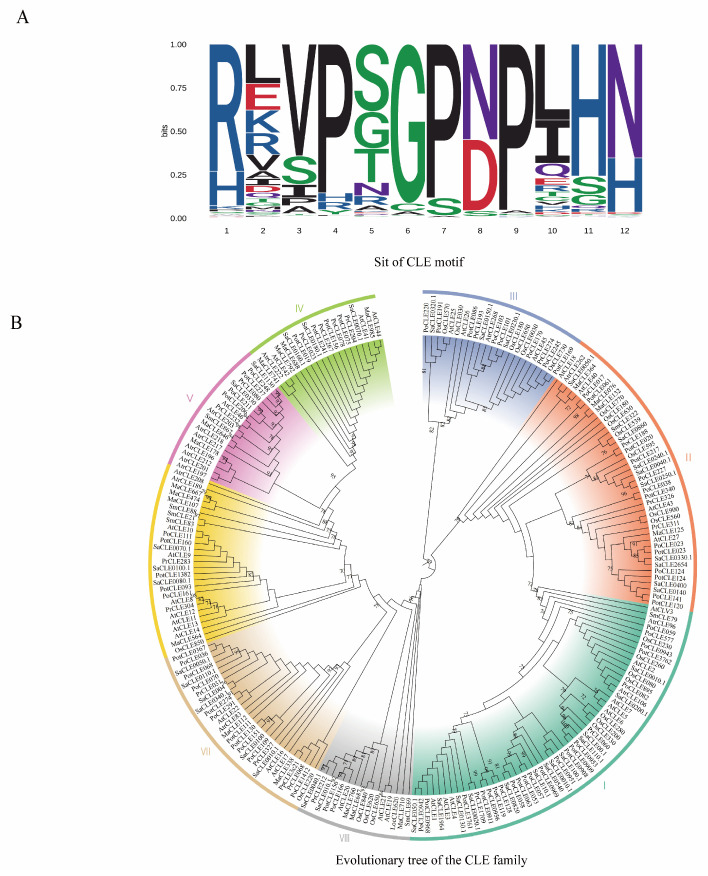
Evolutionary analysis of the CLE family in multiple species. (**A**) Select 10 representative species spanning from lower to higher organisms and construct a CLE motif map based on the conserved regions of 12 amino acids. (The protein sequences were obtained from NCBI (Supplementary Table S1) and draw the CLE motif map by using the online software webLogo 3 (Version 2.8.2) (http://weblogo.berkeley.eduMogo.cgi)) accessed on 10 January 2025. (**B**) Phylogenetic tree analysis of CLE protein families. Relationship of CLE proteins with homologs from other important plant species was constructed using the MEGA 11 program, after aligning the protein sequences with MUSCLE. accessed on 15 January 2025. The phylogenetic tree analysis revealed distinct clusters, denoted as Cluster I, Cluster II, Cluster III, Cluster IV, Cluster V, Cluster VI, Cluster VII, and Cluster VIII.

When we review the research process of the CLE family, it is not difficult to find that although the CLE family has continuously evolved over millions of years, and in addition to parasitic nematodes, the CLE family is found in plants and is one of the largest families of expanded plant polypeptides [16,169] (Figure 2A). However, reports-of-CLE-in-non-pattern. woody plants are very limited, mainly for the following reasons.

As non-flowering woody plants continue to evolve, it remains unclear whether the CLE family have evolved into more refined branches, which seriously affects the search for *CLE* family homologous genes in trees. Here, we conduct extensive studies on mosses (such as *P. patens* [163]), ferns (such as *S. moellendorffii*), gymnosperms (such as *P. abies*), and angiosperms (such as *O. sativa* [170], *Arabidopsis* [11], *P. trichocarpa* [37] *S. purpurea*, *P. deltoides*, *P. persica*, *A. trichopoda* [171] (Figure 2B). We found that the CLE family between lower plants and higher plants has changed significantly, and more complex and precise branches have been differentiated. However, the amino acid structure of CLE was still conserved (Figure 2A). Therefore, we can use the research methods in *Arabidopsis thaliana*, such as molecular probes and gene editing, to locate the *CLE* gene family in forest trees.

**Figure 3:**
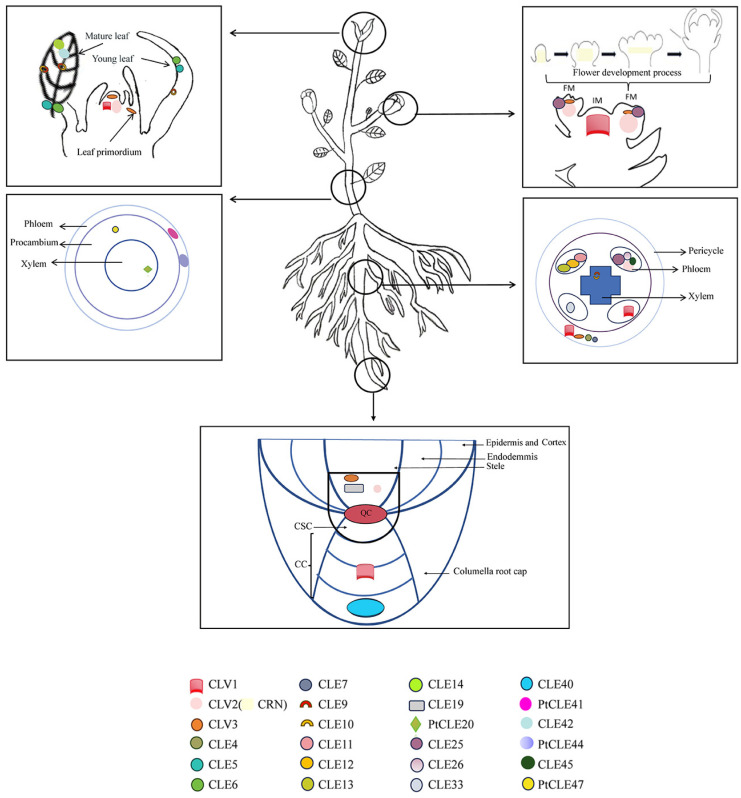
The position where the CLE family performs its own functions throughout the development of Meristem in plants. The stems apical meristems of the plant (including those of leaves), flower meristems, cambium (both stem and root cambium) and root apical meristems are marked and presented in vertically arranged box-like structures from top to bottom. Different colors and shapes were used to mark the CLE family in positions of the plant.

The tissue positioning of the *CLE* family during plant development in woody plants is currently in a very unclear state. Most woody plants are also limited to only one of the last few genes in the *CLE* family and are researchers unable to form an overall network structure. Our analysis revealed overlapping expression profiles of *CLE* gene family in diverse meristematic tissues, such as *CLV1* being expressed in roots, stems, and cambium (Figure 3). *CLV1*/*CLV2*/*CRN* functions as a signaling transduction component extensively involved in plant organ developmental processes. This study shows that receptor kinases exhibit multi-organ distribution characteristics. The existence pattern of receptor kinase is highly conserved. It is very likely that the *CLE* gene family in woody plants are also located in the tissues of woody plants. For specifically expressed genes like *CLV3*, their regulatory pathways demonstrate shared features in root and shoot tissues (Figure 3). Based on evolutionary conservation analysis of the *CLE* family, *CLV3* orthologous genes likely exert regulatory roles in both shoot and root apical meristems of woody plants. Comparative analysis of root and shoot procambium tissues revealed no functional redundancy among CLE family members such as CLE25 and PtCLE20 (Figure 3). These findings indicate that the *CLE* family exhibits both specific single-gene expression patterns and potentially unidentified functionally related genes. This suggests that CLE peptides can function both as individual initiation signals and as signaling molecules that coordinate with other genes to regulate plant development. This unique genetic function provides a very high degree of accuracy for the research on the cambium of woody plants.

The trees passed down from generation to generation are large in size and come in various shapes. The developmental regulatory networks existing in plant organs and tissues are far more complex than those in some model plants. Although *Arabidopsis thaliana* confers limited translational applicability for arboreal species, developmental genetic analyses of meristem regulatory networks have established that a phloem-specific CLE41-PXY/TDR-WOX4 regulatory circuit in *Populus trichocarpa* is discovered based on the WUS-CLV3 ligand–receptor module that is an evolutionarily conserved regulatory module (Figure 1). This suggests that the reference *Arabidopsis* regulatory network is crucial for elucidating the CLE family foundation in forest trees. This necessitates synergistic integration of pan-omics analyses (spatiotemporal proteomics, phospho-signaling mapping) with CRISPR-Cas9-mediated tissue-specific *CLE* knockout systems to resolve the mechanistic coupling between peptide ligand gradients and xylary differentiation trajectories in woody perennials. For floral and foliar organs in forest plants, auxin regulation could serve as a key entry point to elucidate the CLE signal transduction network in forest trees. Furthermore, it is imperative to integrate additional biological experiments to advance this research and address existing challenges for ultimately enhancing wood yield.

The *CLE* family involves more sophisticated compensation mechanisms during plant development. Not only for trees, but for all plants, there is even more profound room for the study of this compensation mechanism. The investigation into the compensation mechanism of the *CLE* gene family has revealed functional complementarity among its members. This signaling compensation fundamentally underpins meristem homeostasis, wherein developmental robustness is achieved via multilayered feedback control rather than isolated genetic components. Given this systems-level complexity, reductionist approaches focusing on single-gene characterization fail to capture the gene function. Therefore, it is imperative to explore diverse methodologies for a more scientific and comprehensive understanding of genes involved in forest tree development, such as protein interactome mapping, genome sequencing, and so on. The mechanistic insights derived from such multidimensional analyses hold significant potential for optimizing genome-informed silvicultural practices aimed at enhancing carbon sequestration efficiency and ecosystem service provisioning in managed forest stands.

In summary, as members of the polypeptide family, the *CLE* family genes act as signal regulators in meristems, maintaining the balance and transformation of stem cell homeostasis and thereby exerting regulatory effects on plant growth and development. Although extensive research has been conducted on model plants such as *Arabidopsis thaliana* and herbaceous plants, research on large, long-lived trees that play a key role in climate regulation and ecological balance is still limited. Therefore, it is more important to expand the research on how the CLE family regulates the meristems of forest trees.

## Data Availability

Data are contained within the NCBI.

## Supplementary Materials

The following supporting information can be downloaded at: https://www.mdpi.com/article/10.3390/plants14101424/s1, Table S1: multispecies CLE motif map.

## Abbreviations

The following abbreviations are used in this manuscript:

CLAVATA 1	CLV1	CLE7	CLV3/ESR1-LIKE7	CLE14	CLV3/ESR1-LIKE14	CLE40	CLV3/ESR1-LIKE 40
CLAVATA 2	CLV2(CRN)	CLE9	CLV3/ESR1-LIKE9	CLE19	CLV3/ESR1-LIKE19	PtCLE41	Populus trichocarpa CLV3/ESR1-LIKE41
CLV3	CLAVATA3	CLE10	CLV3/ESR1-LIKE10	PtCLE20	Populus trichocarpa CLV3/ESR1-LIKE20	PtCLE42	Populus trichocarpa CLV3/ESR1-LIKE42
CLE4	CLV3/ESR1-LIKE4	CLE11	CLV3/ESR1-LIKE11	CLE25	CLV3/ESR1-LIKE25	PtCLE44	Populus trichocarpa CLV3/ESR1-LIKE44
CLE5	CLV3/ESR1-LIKE5	CLE12	CLV3/ESR1-LIKE12	CLE26	CLV3/ESR1-LIKE26	CLE45	CLV3/ESR1-LIKE45
CLE6	CLV3/ESR1-LIKE6	CLE13	CLV3/ESR1-LIKE13	CLE33	CLV3/ESR1-LIKE33	PtCLE47	Populus trichocarpa CLV3/ESR1-LIKE47
FAS1/FAS2	FASCIATA1 and FASCIATA1	STM	SHOOT MERISTEMLESS	WUS	WUSCHEL	TDIF	tracheary element differentiation inhibitory factor
RAM	Root Apical Meristem	SCN	stem cell niche	QC	quiescent center	CSC	columella stem cells
CC	columella cells	CIKs	CLAVATA3 INSENSITIVE RECEPTOR KINASES	ACR4	ARABIDOPSIS CRINKLY4	WOX5	WUS-RELATED HOMEOBOX5
SAN	Shoot Apical Meristem	OC	organizing center	PZ	peripheral zone	RM	rib meristem
HAM	HAIRY MERISRTEM	LRR-RLKs	LEUCINE-RICH REPEAT RECEPTOR-LIKE KINASES	SOL2	SUPPRESSOR OF LLP1 2	PM	plasma membrane
ER	endoplasmic reticulum	ER	the transmembrane	BAM	BARELY ANY MERISTEM	POL	POLTERGEIST
RPK2	RECEPTOR-LIKE PROTEIN KINASE2	PC	procambium	PPh	protophloem	Pxy	protoxylem
MC	metacambium	TDR/PXY	TDIF RECEPTOR/PHLOEM INTERCALATED WITH XYLEM	GSK3s	GLYCOGEN SYNTHASE KINASE 3 PROTEINS	BES1	BRI1-EMS SUPPRESSOR 1
VCM2/PIN5	VASCULAR CAMBIUM-RELATED MADS2/PIN-FORMED5	CLERK-CLV2	TOR KINASE -CLV2	MMCs	meristemoid mother cells	PIN1	PIN-FORMED1
TEs	tracheary elements	BRX	BREVIS RADIX	RPK2	RECEPTOR-LIKE PROTEIN KINASE2	SEs	sieve element
ARFs	AUXIN RESPONSE FACTORS	BRM	BRAHMA	ESR1/DRN	ENHANCER OF SHOOT REGENERATION1/DORNROSCHEN	MAPK	MEMBRANE-ASSOCIATED KINASE REGULATOR 5
HSL1	HAESA-LIKE1	AM	axillary meristem	ACS	THE ENZYME 1-AMINOCYCLOPROPANE-1-CARBOXYLIC ACID SYNTHASE	EBF1/2	ETHYLENE-INSENSITIVE3 (EIN3)-binding F-BOX1/2
EIN3	ETH-YLENE-INSENSITIVE3	FM	Floral Meristem	IM	inflorescence meristem	PLL1	POL-LIKE1
TD1	THICK TASSEL DWARF	ERF2	FASCIATED EAR2	YUC	YUCCA	CLE42	CLV3/ESR1-LIKE42

Additionally, because there are many *Arabidopsis* species gene names involved in this paper, we default to not adding prefixes, and other species *CLE* gene families add abbreviations before the species.
